# Exploring surface structures

**DOI:** 10.7554/eLife.96485

**Published:** 2024-02-28

**Authors:** Bernhard Schuster

**Affiliations:** 1 Institute of Synthetic Bioarchitectures, Department of Bionanosciences, University of Natural Resources and Life Sciences, Vienna Vienna Austria

**Keywords:** archaea, Sulfolobus, S-layer, cryoEM, tomography, single-particle analysis, Other

## Abstract

The surface layer of *Sulfolobus acidocaldarius* consists of a flexible but stable outer protein layer that interacts with an inner, membrane-bound protein.

**Related research article** Gambelli L, McLaren M, Conners R, Sanders K, Gaines MC, Clark L, Gold VAM, Kattnig D, Sikora M, Hanus C, Isupov MN, Daum B. 2024. Structure of the two-component S-layer of the archaeon *Sulfolobus acidocaldarius*. *eLife* **13**:e84617. doi: 10.7554/eLife.84617.

Archaea and bacteria have much in common: both are single-celled microorganisms, and neither has a nucleus – so they are both prokaryotes. Archaea are also found in all the niches inhabited by bacteria. However, archaea can also survive in extreme niches where bacteria cannot. Most archaea live in very cold conditions, but they can also live in hot springs, or near deep-sea vents where temperatures can exceed 100 degrees Celsius, or in the extremely high pressures found at the bottom of the ocean. Other archaea can survive in conditions that are extremely saline, alkaline or acidic (down to pH 0), and some can even thrive in petroleum deposits deep underground.

How can archaea survive in these environments? And how, in particular, can archaeal cells withstand the extremes of temperature, pressure, salinity and pH that they are subjected to? The cell envelope in a prokaryote includes a cell wall that provides structural integrity, and a membrane that encloses the cytoplasm of the cell. There are important differences in the constituents and construction of the cell wall in archaea and bacteria, but there are also similarities, notably the presence in almost all archaea, as well as many bacteria, of a two-dimensional lattice called a surface layer (Sleytr et al., 1988; Bharat et al., 2021). These layers are made of subunits called surface-layer proteins (SLPs), and unlike what happens in bacteria, the surface layer in archaea – with just a few exceptions – must interact with the cytoplasmic membrane (Albers and Meyer, 2011; Rodrigues-Oliveira et al., 2017). Moreover, prokaryotes must synthesize, translocate to the cell surface, and incorporate into the existing lattice at least 500 copies of each SLP every second to maintain the surface layer (Sleytr et al., 1999).

In some archaea the surface layer is made of two different SLPs, although only one of these need interact with the cytoplasmic membrane. However, there is much about two-component surface layers that we do not fully understand. Now, in eLife, Bertram Daum from the University of Exeter and co-workers – including Lavinia Gambelli as first author – report details of an in situ atomic model of a two-component surface layer that sheds new light on the dynamics and assembly of these structures (Gambelli et al., 2024). The study was performed with samples from *Sulfolobus acidocaldarius*, an archaeal species that lives in hot springs, and relied on a combination of experimental techniques – notably cryo electron microscopy and cryo electron tomography – and a software package called Alphafold2 that predicts protein structures.

The surface layer in *S. acidocaldarius* is made of two proteins: SlaA is a Y-shaped soluble protein rich in β-strands, while SlaB contains three consecutive β-sandwich domains and a membrane-bound coiled-coil domain at its C-terminus (Figure 1A and B). In previous work Gambelli et al. had shown that the unit cell of the surface layer was hexagonal and contained three dimers of SlaA and a trimer of SlaB (Figure 1C; Gambelli et al., 2019).

**Figure 1.**
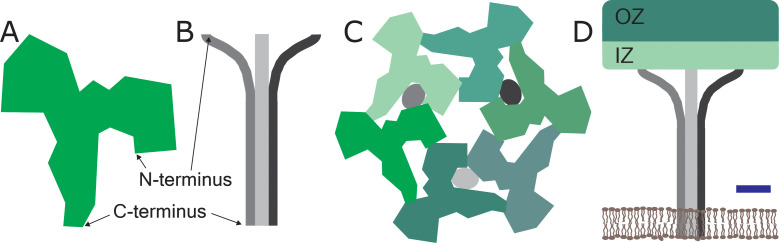
The structure of a two-component surface layer in the cell envelop of the archaeal species *Sulfolobus acidocaldarius*. The surface layer of *S. acidocaldarius* is made of two glycosylated proteins: SlaA, which is extracellular-facing and is shown here as a monomer (**A**), and SlaB, which is intracellular-facing and is shown here as a trimer (**B**): the glycosylation is not shown for either protein. (**C**) Schematic extracellular view of the unit cell of the surface layer, which contains three SlaA dimers (with the six monomers shown in different shades of green) and the SlaB trimer (with the monomers shown in shades of grey). The SlaA dimers assemble around a central, hexagonal pore. This also gives rises to a ring of six triangular pores, three of which are occupied by the SlaB trimer. (The trimer occupies the pores between adjacent dimers, and not the pores within the dimers). (**D**) Schematic side view of one unit cell. The SlaA dimers and the SlaB trimer create a canopy-like framework that is roughly parallel to the cytoplasmic membrane (shown in brown). Each SlaA protein contains six domains: the four domains nearest the N-terminus form the outermost surface, which is called the outer zone (OZ), and the other two domains project towards the cytoplasmic membrane and form the inner zone (IZ). The N-terminus of each monomer in the SlaB trimer interacts with the SlaA lattice, while the transmembrane domain at the C-terminus of each monomer anchor the lattice to the cytoplasmic membrane. Scale bar: 5 nm.

Now they show that the SlaA dimers assemble into a sheet with a thickness of 9.5 nm, and that the individual proteins adopt an angle of about 28° with respect to the plane of the cytoplasmic membrane. This sheet is anchored to the cytoplasmic membrane by the SlaB trimers – which have their long axes perpendicular to the SlaA sheet– to create a canopy-like framework with an overall thickness of 35 nm (Figure 1D). One of the reasons why the SlaA sheet is robust is because the SlaA proteins have formed dimers. However, there is also some flexibility in the structure because two of the six domains in each SlaA protein – the two domains nearest the C-terminus – do not adopt fixed positions, and are thus free to move to some extent.

Surface layers have already shown potential for applications in biotechnology, medicine and environmental science, and an improved understanding of these structures could lead to further applications in fields as diverse as ultrafiltration membranes and biosensors (Pfeifer et al., 2021; Pfeifer et al., 2022; Douglas et al., 1986). These applications in the real world are a long way from the extreme environments in which archaea are often found.
